# The impact of a human resource management intervention on the capacity of supervisors to support and supervise their staff at health facility level

**DOI:** 10.1186/s12960-017-0225-0

**Published:** 2017-08-30

**Authors:** Ogenna Uduma, Marie Galligan, Henry Mollel, Honorati Masanja, Susan Bradley, Eilish McAuliffe

**Affiliations:** 1 Centre for Global Health, Trinity College Dublin, Dublin, Ireland; 2 School of Health Sciences, City University London, 1 Myddelton Street, London, EC1R 1UW United Kingdom; 3 Ifakara Health Institute, Dar es Salaam, Tanzania; 4 School of Medicine, University College Dublin, Dublin, Ireland; 5 School of Nursing, Midwifery and Health Systems, University College Dublin, Dublin, Ireland

**Keywords:** Supportive supervision, Supervisors, Intervention, Supervision

## Abstract

**Background:**

A systematic and structured approach to the support and supervision of health workers can strengthen the human resource management function at the district and health facility levels and may help address the current crisis in human resources for health in sub-Saharan Africa by improving health workers’ motivation and retention.

**Methods:**

A supportive supervision programme including (a) a workshop, (b) intensive training and (c) action learning sets was designed to improve human resource management in districts and health facilities in Tanzania. We conducted a randomised experimental design to evaluate the impact of the intervention. Data on the same measures were collected pre and post the intervention in order to identify any changes that occurred (between baseline and end of project) in the capacity of supervisors in intervention a + b and intervention a + b + c to support and supervise their staff. These were compared to supervisors in a control group in each of Tanga, Iringa and Tabora regions (*n* = 9). A quantitative survey of 95 and 108 supervisors and 196 and 187 health workers sampled at baseline and end-line, respectively, also contained open-ended responses which were analysed separately.

**Results:**

Supervisors assessed their own competency levels pre- and post-intervention. End-line samples generally scored higher compared to the corresponding baseline in both intervention groups for competence activities. Significant differences between baseline and end-line were observed in the total scores on ‘maintaining high levels of performance’, ‘dealing with performance problems’, ‘counselling a troubled employee’ and ‘time management’ in intervention a + b. In contrast, for intervention a + b + c, a significant difference in distribution of scores was only found on ‘counselling a troubled employee’, although the end-line mean scores were higher than their corresponding baseline mean scores in all cases. Similar trends to those in the supervisors’ reports are seen in health workers data in terms of more efficient supervision processes, although the increases are not as marked.

**Conclusion:**

A number of different indicators were measured to assess the impact of the supportive supervision intervention on the a + b and a + b + c intervention sites. The average frequency of supervision visits and the supervisors’ competency levels across the facilities increased in both intervention types. This would suggest that the intervention proved effective in raising awareness of the importance of supervision and this understanding led to action in the form of more supportive supervision.

**Electronic supplementary material:**

The online version of this article (doi:10.1186/s12960-017-0225-0) contains supplementary material, which is available to authorized users.

## Background

There is a growing body of evidence on the importance of Human Resource Management (HRM) in the quality of services that health workers are able to deliver. Agencies such as the Capacity Project have identified planning, developing and supporting the workforce as the three pillars needed to strengthen human resources for health (HRH) to implement quality health programming in developing countries [1]. Most governments are addressing the planning aspects of HRM, through initiatives such as increasing the output from health professional training colleges or attracting retired health workers back into the workforce, but outputs from previous research [2] clearly articulate the need to address support of the workforce.

Supporting the workforce involves strengthening systems to improve and sustain health worker performance. Central to this is the critical need to improve supervision systems to enhance health worker motivation and productivity. Sporadic supervision visits from district management and programme managers have been found to be erratic and their use of conflicting guidance during supervision leaves staff confused and demoralized [3]. The World Health Organization [4] emphasized that supportive supervision that strengthens relationships, identifies/resolves problems and gives constructive feedback can contribute more to performance of health workers; however, the operationalization of the supportive supervision concept is challenging. In many resource-constrained countries, the traditional visiting supervisor model is common, but there is broad consensus that this is not effective [3, 5]. Benefits of having supportive supervisors in the workplace include enhanced job performance, satisfaction, collaboration and organizational commitment for subordinates, as well as reduced turnover intentions [6–9]. Ongoing support is needed for health workers in the frontline of service delivery to perform to their full potential. Supervision, therefore, is one of the most relevant tasks in HRM. However, health managers commonly neglect supervision, and many supervisors lack the knowledge, skills and tools for effective supervision [10].

Tanzania’s Ministry of Health and Social Welfare (MOHSW) has made several efforts to address supervision at the primary care level by structuring the process to ensure that every frontline staff member is supervised [10]. Primary health care workers in Tanzania have reported dissatisfaction with the supervision they receive and often go months without supervision [11]. The quality of the supervisory visits also vary, though many found it helpful it was reported to be infrequent and of poor quality because of the minimal amount of time spent at the facilities during supervision [12]. Supervisors were also reported to be judgemental, fault finding and unsystematic in their approach and to provide inadequate feedback [13]. Reviewing the literature and current developments in Tanzania human resource training, it has become apparent that what is needed is a comprehensive, feasible, methodologically sound and evaluated intervention to improve HRM.

The Support, Train and Empower Managers (STEM) intervention was designed to provide a structure and skill set to put HRM policies into practice by supporting health workers in supervisory roles to provide supportive supervision. This paper reports changes in supervisors’ assessment of their competency levels as a result of taking part in the study.

## Methods

### Study setting

The study took place in the regions of Iringa, Tanga and Tabora in Tanzania. The selection of the districts was influenced by the team’s decision to:

- Work with facilities where few or no other non-governmental or governmental organisations were currently providing supervision training
- Include a range of facilities with high, medium and low levels of attractiveness as places to work (this was determined through analysis of data from a previous study)
- Include geographically dispersed councils to reduce the risk of cross-contamination between groups that might be located in neighbouring councils

In each region, three districts were selected with two intervention groups and one control group, i.e. Handeni, Muheza and Tanga city (Tanga region), Ludewa, Mufindi and Iringa Municipal (Iringa region) and Urambo, Nzega and Tabora municipal (Tabora region). Five health facilities were selected for inclusion in the study from each district and were assigned to the same group. The criteria for selection were facility level and staffing level. Regarding facility level, all health centre in the district were first selected (all have a larger number of staff compared to dispensaries), and where the number health centre is lower than required, the dispensaries that provide Reproductive Child Health Services (RCH) and with higher staffing level compared to others in the district were selected to fulfil the requirement. All health workers in the selected districts participated in the study. A total of 45 health facilities were sampled in Tanzania, 15 from each of three geographical regions—Iringa, Tabora and Tanga at baseline and end-line. Within each region, five health facilities were sampled from each of three districts. The sampled health facilities included both health centres (21) and dispensaries (24). Additional file 1: Table S1 shows the distribution of sampled health centres and dispensaries across the three regions, grouped by district and intervention group. All five health facilities in each of the selected councils were assigned to the same group or cluster.

The intervention components were (a) workshop with district health management teams and facility managers on human resource management, (b) intensive training in supervisory and support skills for managers directly engaged in supervision, aimed at strengthening the capacity of these in-charges at a facility level or (c) action learning sets for staff engaged in supervision at the district and facility level which followed on from the training and continued for a period of 12 months.

The first element of the STEM was a series of workshops on HRM with the reproductive and child health personnel on the District Health Management Team and with obstetric care facility managers; focus group discussions were also carried out with these personnel. The data generated from the workshop and focus group discussions provided us with information on their needs and priorities.

The second element was a 5-day training with health workers with supervisory responsibility and district level staff with supervisory responsibilities. The challenges and practice gaps that supervisors had highlighted during the workshop were used to refine and focus the content of the training. They were also used as the foundation for some exercises or peppered throughout the training to illustrate key learning points, bringing texture and clarity to the theory. The training content and implication is being reported in another paper. This intensive training aimed to equip supervisors with the knowledge, skills and attitudes necessary to undertake effective, supportive supervision. The complete set of training slides and facilitator’s notes can be found in the STEM Training Manual for Facility Supervisors. The training was designed to challenge participants to rethink the elements of their current supervision process. Its focus on supportive supervision aimed to engender a mindset where teams of health workers identified their own challenges and achieved results with support from their supervisors.

The third element was action learning sets with supervisors in the facilities selected for the intervention which met monthly over a period of 1 year. The ALS built on the learning from the intensive training and allowed participants to discuss successes and challenges in implementing their new supervisory skills; it was also aimed to support collaborative learning. The ALS enabled supervisors to participate in a peer group network who met once a month to provide mutual learning and support and to share best practices. Results of the impact of the ALS are reported elsewhere.

The overall objective of the STEM project (support, train and empower managers) was to strengthen the human resource management (HRM) function at district and health facility level, by increasing the capacity of managers to support and supervise their staff. The study was based on two hypotheses.

1. Regular supportive supervision delivered by local facility staff will improve job satisfaction and engagement and reduce turnover
2. Action learning sets will enhance the anticipated benefits of supportive supervision.

This paper reports only on the perceptions of supervisors and health workers in changes of the supervision competency level at the target facilities before and after the intervention. Other aspects of the study including the action learning sets, impact on health workers are reported in separate papers. We used a quasi-experimental design with districts assigned to intervention or control groups and pre- and post-test measures were taken from supervisors and health workers to evaluate the impact of the following interventions on HRM:

- Group 1: Intervention with steps a and b—a + b
- Group 2: Intervention with steps a, b, and c—a + b + c
- Group 3: No intervention—control.

### Data collection

The baseline study assessed supervisors’ (direct beneficiaries) and health workers (indirect beneficiaries) understanding of and competence in supervisory tasks, as well as the frequency with which they perform the key tasks of supervision. Data were collected on the same measures at the end of the project to identify any changes that occurred (between baseline and end of project) in the capacity of managers/supervisors in intervention group 1 (a + b) and group 2 (a + b + c) to support and supervise their staff, and compare these to managers/supervisors in control group 3.

The baseline data collection took place in May and June 2012 and end-line data collection took place in March and April 2014, with the intervention components running from June/July 2012 to February 2014. The sample of respondents comprised approximately 40% of the total available staff in the targeted facilities on the day of data collection for baseline and 38% for end-line.

The profile of supervisors at baseline and end-line was similar although end-line sample has a slightly lower average age; there were a total of 95 and 108 supervisors sampled at baseline and end-line respectively. Demographic characteristics were broadly similar in the two samples. Forty-one supervisors completed both baseline and end-line surveys (10 control, 18 intervention a + b and 13 intervention a + b + c). Table 1 shows the breakdown of sample size by intervention group, which is similar at baseline and end-line. The health worker sample distributions across intervention groups were also similar at baseline and end-line, with a larger percentage of health workers from intervention a + b + c districts (46.4% at baseline, 42.8% at end-line). The control group had the lowest relative sample size, making up about one fifth of the baseline and end-line samples. A total of 196 health workers sampled at baseline and 187 at end-line (Table 2). Thirty-two health workers completed both the baseline and end-line questionnaires (4 control, 9 intervention a + b and 19 intervention a + b + c).

**Table 1:** Demographics of Supervisors

Stage	Baseline			Endline		
Intervention group	Control	A + B	A + B + C	Control	A + B	A + B + C
Number of supervisors	27	32	36	27	40	41
Gender						
Male	12 (44.4%)	10 (31.2%)	11 (30.6%)	12 (44.4%)	10 (25%)	12 (29.3%)
Female	15 (55.6%)	22 (68.8%)	25 (69.4%)	15 (55.6%)	30 (75%)	29 (70.7%)
Age in years						
Mean (sd)	46.9 (8.7)	43.9 (10.5)	41.1 (8.5)	40.4 (11.4)	41.4 (10.8)	40.7 (10.1)
Highest medical or paramedical qualification						
Medical Officer	2 (7.4%)	0 (0%)	1 (2.8%)	2 (7.4%)	1 (2.5%)	1 (2.4%)
Assistant Medical Officer	1 (3.7%)	4 (12.5%)	3 (8.3%)	1 (3.7%)	4 (10%)	5 (12.2%)
Clinical officer	9 (33.3%)	7 (21.9%)	12 (33.3%)	4 (14.8%)	10 (25%)	9 (22%)
Assistant clinical officer	1 (3.7%)	1 (3.1%)	2 (5.6%)	3 (11.1%)	2 (5%)	3 (7.3%)
Medical attendant	2 (7.4%)	1 (3.1%)	1 (2.8%)	3 (11.1%)	0 (0%)	2 (4.9%)
Registered nurse	3 (11.1%)	6 (18.8%)	12 (33.3%)	7 (25.9%)	11 (27.5%)	12 (29.3%)
Registered nurse midwife	3 (11.1%)	7 (21.9%)	3 (8.3%)	3 (11.1%)	7 (17.5%)	5 (12.2%)
Enrolled nurse	2 (7.4%)	2 (6.2%)	1 (2.8%)	0 (0%)	3 (7.5%)	3 (7.3%)
Enrolled nurse midwife	3 (11.1%)	3 (9.4%)	1 (2.8%)	2 (7.4%)	0 (0%)	0 (0%)
MCH Aides	1 (3.7%)	1 (3.1%)	0 (0%)	2 (7.4%)	2 (5%)	1 (2.4%)
Current job position						
Facility incharge	12 (44.4%)	11 (34.4%)	15 (41.7%)	8 (29.6%)	12 (30%)	15 (36.6%)
Assistant facility incharge	1 (3.7%)	1 (3.1%)	2 (5.6%)	3 (11.1%)	4 (10%)	5 (12.2%)
Head of unit	10 (37%)	14 (43.8%)	13 (36.1%)	10 (37%)	14 (35%)	16 (39%)
Head of department	4 (14.8%)	4 (12.5%)	6 (16.7%)	1 (3.7%)	2 (5%)	0 (0%)
Other	0 (0%)	2 (6.2%)	0 (0%)	5 (18.5%)	8 (20%)	5 (12.2%)

**Table 2:** Demographics of Health workers

Stage	Baseline			Endline		
Intervention group	Control	A + B	A + B + C	Control	A + B	A + B + C
Number of health workers	43	62	91	38	69	80
Gender						
male	13 (30.2%)	15 (24.6%)	13 (14.3%)	12 (31.6%)	13 (19.1%)	9 (11.2%)
female	30 (69.8%)	46 (75.4%)	78 (85.7%)	26 (68.4%)	55 (80.9%)	71 (88.8%)
Age in years						
Mean (sd)	44 (10)	40 (10)	39 (10)	36 (10)	37 (11)	36 (11)
Highest medical or paramedical qualification						
Medical Officer	0 (0%)	0 (0%)	0 (0%)	0 (0%)	0 (0%)	1 (1.2%)
Assistant Medical Officer	1 (2.3%)	0 (0%)	3 (3.3%)	0 (0%)	0 (0%)	1 (1.2%)
Clinical officer	3 (7%)	7 (11.3%)	1 (1.1%)	0 (0%)	3 (4.3%)	0 (0%)
Assistant clinical officer	0 (0%)	3 (4.8%)	6 (6.6%)	0 (0%)	4 (5.8%)	3 (3.8%)
Medical attendant	22 (51.2%)	34 (54.8%)	42 (46.2%)	19 (50%)	21 (30.4%)	35 (43.8%)
Registered nurse	5 (11.6%)	4 (6.5%)	17 (18.7%)	10 (26.3%)	20 (29%)	17 (21.2%)
Registered nurse midwife	3 (7%)	6 (9.7%)	7 (7.7%)	3 (7.9%)	11 (15.9%)	11 (13.8%)
Enrolled nurse	3 (7%)	3 (4.8%)	9 (9.9%)	2 (5.3%)	2 (2.9%)	5 (6.2%)
Enrolled nurse midwife	4 (9.3%)	1 (1.6%)	4 (4.4%)	1 (2.6%)	5 (7.2%)	3 (3.8%)
MCH Aides	1 (2.3%)	1 (1.6%)	1 (1.1%)	3 (7.9%)	3 (4.3%)	4 (5%)
Laboratory staff	1 (2.3%)	3 (4.8%)	1 (1.1%)	0 (0%)	0 (0%)	0 (0%)
Number of years working at health facility						
Mean (sd)	8.8 (8.9)	8.7 (9)	2.9 (2.6)	4 (4.5)	7.6 (7.4)	3.5 (2.7)
Current job position						
facility incharge	0 (0%)	0 (0%)	0 (0%)	0 (0%)	1 (1.4%)	1 (1.2%)
assistant facility incharge	2 (4.7%)	3 (4.8%)	3 (3.3%)	1 (2.6%)	2 (2.9%)	2 (2.5%)
head of unit	0 (0%)	0 (0%)	0 (0%)	1 (2.6%)	2 (2.9%)	2 (2.5%)
head of department	1 (2.3%)	3 (4.8%)	2 (2.2%)	0 (0%)	1 (1.4%)	0 (0%)
other	40 (93%)	56 (90.3%)	86 (94.5%)	36 (94.7%)	63 (91.3%)	75 (93.8%)

Prior to data collection, meetings were held with the key stakeholders, i.e. HR personnel in Ministry of Health, District Health Personnel, Development Partners and NGOs working on strengthening the human resource management in each country’s health system, as well as Irish Aid development specialists and Heads of Mission in Tanzania. Each of the nine selected councils was informed about tentative dates for data collection. As such, the relevant staff (the District Health Secretary, District Medical Officer (DMO), and District Reproductive and Child Health (RCH) Coordinator) at the council level were aware and prepared to receive the research team. This facilitated planning and logistical arrangements for data collection. In every district, data collection began after a brief introduction of the STEM team to the relevant council top officials, i.e. the District Executive Director, District Medical Officer and District Health Secretary. The introduction included seeking facilitation of the teams to the selected health facilities. All data collection was carried out using standard ethical procedures. The objectives of the study were explained and confidentiality was assured. Informed, signed consent was obtained from every respondent and all data and records were rendered anonymous through the use of a unique identity number.

The STEM intervention aimed to change the behaviour of supervisors and having a positive impact on health workers’ experience of supervision and on their levels of job satisfaction and engagement as a result of positive changes in the behaviour of their supervisors so needed instruments that captured supervisor performance and perspectives. This was achieved by adapting an existing Supervisor Competency Self-Assessment Inventory developed by Management Sciences for Health. This is a self-report measure designed to assess the frequency with which in-charges/managers carry out 24 specific supervisory activities. The inventory was administered to health facility personnel in all project districts who had supervisory responsibility (e.g. facility in-charge, maternity/ward in-charge, programme supervisors). The questionnaire has been used by individuals and organisations to assess their competence and performance as supervisors and the results used to develop a plan of improvement. It is also used as a guide to curriculum development for supervisory training using the components as a basis for a need assessment exercise [14].

Section A contains demographics, medical qualifications, current job position and length of service in the current facility.

Section B contains information on the manager’s experience of supervision. It includes the number of staff for whom they have supervisory responsibility, and the frequency, type and duration of supervision used.

Section C is the self-assessment inventory. It asks participants how frequently they perform a range of supervisory activities. Response options are never (0%), rarely (less than 25%), sometimes (25–75%), often (more than 75%) and always (100%). Supervisory activities are grouped into five clusters:

- Interactions with my staff
- Maintaining high levels of performance
- Dealing with performance problems
- Counselling a troubled employee
- Time management

Section D consists of three open-ended questions on how to support staff performance.

Section E consists of changes in supervisory activities since the introduction of STEM.

Section F consists on usefulness and impacts of STEM Diary.

Section G consists on usefulness and impact of STEM Action Learning Sets.

Health workers completed a health worker survey which included information on their demographics (Additional file 2), their experience of supervision, and a corresponding survey to the Supervisor Competency Self-Assessment Inventory (CSAI) to assess health workers’ perceptions of their supervisor’s performance across the same clusters (excluding time management). Items from the supervisor CSAI were slightly re-worded to be assessed from the health worker perspective. For example ‘In my interactions with staff, I communicate my general expectations about performance to staff’ was worded as ‘In interactions with staff, my supervisor let’s me know what is expected of me in my job’. The survey also contained information on health worker job satisfaction, intention to leave burnout and work engagement scale, the results of which are reported elsewhere.

Both surveys (supervisor and health worker) were pre tested in two selected dispensaries in Tanga City which had similar characteristics with the health centres participating in the research. All items proved to be reliable measures, were easily understood by the pilot participants and elicited expected responses in line with previous use [15].

The Supervisor Competency Self-Assessment Inventories were collected in hard copy questionnaires. Responses were then entered into Tablet PCs, using a customised computer application that had been developed for this purpose. These were automatically exported to Excel. An SPSS database was created for storing, cleaning and analysing the data.

### Sample size and statistical power

It was difficult to predict the number of health workers and supervisors that were employed and could be recruited from each facility visited. It was aimed to recruit between 25 and 45 supervisors per treatment group. This would give 80% power to detect an effect size (Cohen’s d) between 0.71 and 0.53 for the CSAI between baseline and end-line (a medium-large effect), at a significance level of 0.05. It was aimed to recruit between 40 and 90 health workers per treatment group, giving 80% power to detect an effect size (Cohen’s d) between 0.56 and 0.37 for the CSAI between baseline and end-line (a small-medium effect), at a significance level of 0.05.

### Data analysis

Data analysis was carried out using R (version 2.15.0), a software environment for statistical computing and graphics. Categorical variables were summarized by frequencies and percentages, while numeric variables were summarized by means and standard deviations. Total scores for each component of the CSAI were calculated by summing over items.

Mann-Whitney *U* tests were used to test for differences in scores on supervision sub-scales between baseline and end-line. The *p* values from these tests were corrected for multiple testing error using the false discovery rate (FDR) approach of Benjamini & Hochberg [16]. Reported are *p* values and adjusted *p* values (FDR-p). Reliability coefficients (Cronbach’s alpha) were calculated in SPSS Version 20.

### Ethical approval

The study was approved by the Health Policy and Management/Centre for Global Health (HPM/CGH) Research Ethics Committee of the Trinity College Dublin (12/002/2011) and the Ifakara Health Institute Institutional Review Board (IHI/IRB/08–2012). Informed, signed consent was obtained from every respondent and all data and records were anonymized by using unique identity numbers.

## Results

### Demographics

Due to constant transfer and rotation of staff in the facilities and districts studied, some of the end-line participants differed from the baseline.

A total of 95 and 108 supervisors were sampled at baseline and end-line respectively. Demographic characteristics were broadly similar in the two samples. Forty-one supervisors completed both baseline and end-line surveys. Table 1 summarizes sample size and demographics by intervention group, and by time point (baseline vs end-line). Demographics are broadly similar across interventions groups and across time points, with some minor differences. Gender distribution was similar at baseline and end-line, but with a slightly higher percentage of males in the control group relative to intervention groups a + b and a + b + c (Table 1). Age distributions were broadly similar, although the end-line sample was a little younger than the baseline sample on average, with an overall mean age of 43.7 at baseline and 40.9 at end-line. The majority of sampled supervisors were clinical officers (29.5% at baseline, 21.3% at end-line) and registered nurses/midwives (35.8% at baseline, 41.7% at end-line). The end-line control group had a lower percentage of clinical officers than other groups, while the baseline control group had a lower percentage of registered nurses/midwives than other groups. Elementary-level cadres (medical attendants/MCH aides, enrolled nurses/midwives) made up 23.2% of the baseline sample and 24.1% of the end-line sample, and there was some variety across intervention groups. The majority of sampled supervisors were facility in-charges (40% at baseline, 32.4% at end-line) and heads of unit (38.9% at baseline, 37% at end-line). Job positions were similar across intervention groups at baseline and end-line. Supervisors (intervention a + b and a + b + c) were asked whether they had ever had any other supervision training or skills building prior to STEM. Three quarters (74.4%) responded that they had not.

A total of 196 health workers were sampled at baseline and 187 at end-line. The distribution of health workers was similar across regions and districts at baseline and end-line. The largest differences in sample size from baseline to end-line were observed in the districts Tabora Urban and Nzega. The sample distributions across intervention groups were also similar at baseline and end-line, with a larger percentage of health workers from intervention a + b + c districts (46.4% at baseline, 42.8% at end-line). The gender distribution of the respondents is similar in baseline at end-line with females in the majority, at 79% of the baseline sample and 81.7% of the end-line sample. There were some differences in gender distribution across intervention groups, the control group having the highest percentage of males and intervention group a + b + c having the lowest. Age distributions of health workers were broadly similar at baseline and end-line although it is evident that the baseline sample (mean age of 40) were a little older on average than the end-line sample (mean age of 36). The age standard deviations were also similar at baseline and end-line. Table 2 shows that the distribution of cadres at baseline and end-line was broadly similar, but with some variations. Medical attendant was the most common cadre in all intervention groups at baseline and end-line. The end-line sample had a higher percentage of registered nurses and midwives (38.5% compared with 21.5% at baseline), a lower percentage of clinical officers (1.6% compared with 5.6% at baseline) and a lower percentage of elementary-level cadres (58.8% compared with 70.2% at baseline). There was also a slight variations in cadre distribution between intervention groups, e.g. a lower percentage of medical attendants in intervention group a + b at end-line.

### Reliability

Cronbach’s alpha was calculated to measure the reliability of the measurement instruments. Reliability was assessed for each of the scales: interactions with staff, maintaining high levels of performance, dealing with performance problems, counselling troubled employees and time management. In the supervisor survey, reliability was at least acceptable (≥0.7) for all scales at baseline and end-line, except for ‘counselling a troubled employee’ at baseline with a Cronbach’s alpha of 0.621 and ‘time management’ at end-line with a Cronbach’s alpha of 0.652 (Additional file 3). In the health worker version of the survey, reliability was good (0.8–0.9) for all scales, and excellent (≥0.9) for ‘maintaining high levels of performance’, at both baseline and end-line.

### Experience of supervision

One-to-one supervision sessions were most common among supervisors interviewed, with 57.9% of baseline supervisors and 47.2% of end-line supervisors responding that this was the most frequent type of supervision session they used.

Supervisor responses about the appropriate duration of supervision were quite different at baseline and end-line. At baseline, 28.4% of supervisors were of the opinion that supervision sessions should last 15–30 min, with around 62% believing supervision sessions should last longer than 30 min. At end-line, a majority of supervisors (57.5%) felt that supervision sessions should last 15–30 min. A similar pattern was observed in supervisors’ responses to ‘How long do YOUR supervision sessions with staff usually last?’. At baseline, over one third of supervisors responded that their supervision sessions usually last 15–30 min, while at end-line this response was provided by 64.8% of supervisors.

Health workers were asked about the frequency, type and duration of supervision they receive from facility in-charges or management. There were some differences between the baseline and end-line responses regarding the frequency of supervision. For example, in the baseline sample there was a lower percentage of health workers reporting that they receive daily supervision from facility in-charges or management (46% compared with 56.1% at end-line), and a higher percentage reporting weekly supervision (31.5% compared with 15.8% at baseline). Responses regarding type of supervision most frequently received from facility in-charges or management were similar between baseline and end-line samples, with approximately half of health workers receiving one-to-one supervision most frequently, and just under a half who most frequently receive supervision in a group/team. Reported usual duration of supervision sessions was similar at baseline and end-line, although there was a higher percentage of health workers at end-line who reported that supervision sessions usually last 15–30 min (53.8% at end-line, 43.5% at baseline) and a lower percentage who reported that the usual duration of supervision sessions with facility in-charges or management was over 30 min.

### Supervisor self-assessment and health worker assessment of supervisors’ performance

Table 3 summarizes the responses of supervisors when asked about their interactions with staff, as a supervisor. In interventions a + b and a + b + c, just 66.2% of supervisors in the baseline sample responded that they usually or always ‘actively attempt to solve problems in the health facility’, while in the end-line sample this percentage was much higher, at 80.2%. Table 14 shows no statistically significant differences in total scores on ‘interactions with staff’ at baseline and end-line, assessed separately for the control, intervention a + b and a + b + c groups.

**Table 3:** Supervisors (intervention a + b and a + b + c) - In interactions with staff…

Item	Baseline (N = 68)			End-line (N = 81)		
	never/rarely	some-times	usually/always	never/rarely	some-times	usually/always
I communicate my general expectations about performance to staff	5 (7.4%)	4 (5.9%)	59 (86.8%)	4 (4.9%)	7 (8.6%)	70 (86.4%)
I listen to staff and am open to their concerns	3 (4.4%)	6 (8.8%)	59 (86.8%)	0 (0%)	7 (8.6%)	74 (91.4%)
I actively attempt to solve problems in the health facility	5 (7.4%)	18 (26.5%)	45 (66.2%)	4 (4.9%)	12 (14.8%)	65 (80.2%)
I treat people fairly and consistently	0 (0%)	3 (4.4%)	65 (95.6%)	0 (0%)	1 (1.2%)	80 (98.8%)
I respect staff and their contributions	0 (0%)	2 (2.9%)	66 (97.1%)	1 (1.2%)	0 (0%)	80 (98.8%)

Table 4 summarizes health workers responses when asked about their supervisor’s interactions with staff. In intervention groups a + b and a + b + c (combined), less than one third (64.7%) of health workers in the baseline sample responded that their supervisor usually or always ‘listens to me and takes notice of my concerns’. At end-line, in contrast, 82.6% of the sample responded that their supervisor usually or always does this (Table 4). Total scores on ‘How my supervisor interacts with staff’ differ a little between baseline and end-line health worker samples (Table 13 and 15). Scores for the control group are lower in general at end-line, are around the same (and a little higher in general) for intervention group a + b, and are generally higher at end-line for health workers in intervention group a + b + c.

**Table 4:** Health Workers (a + b and a + b + c) “In interactions with staff, my supervisor…”

Item	Baseline (N = 153)			End-line (N = 149)		
	never/rarely	sometimes	usually/always	never/rarely	Sometimes	usually/always
Lets me know what is expected of me in my job.	23 (15%)	24 (15.7%)	106 (69.3%)	8 (5.4%)	17 (11.4%)	124 (83.2%)
Listens to me and takes notice of my concerns	25 (16.3%)	29 (19%)	99 (64.7%)	3 (2%)	23 (15.4%)	123 (82.6%)
Tries to take action to solve problems in the facility	21 (13.7%)	20 (13.1%)	112 (73.2%)	2 (1.3%)	15 (10.1%)	132 (88.6%)
Treats people fairly and consistently	11 (7.2%)	14 (9.2%)	128 (83.7%)	4 (2.7%)	7 (4.7%)	138 (92.6%)
Respects staff and their contributions	11 (7.2%)	12 (7.8%)	130 (85%)	2 (1.3%)	7 (4.7%)	140 (94%)

When asked about activities carried out to maintain high levels of performance (Table 5), at baseline, supervisors seemed to have difficulty with ‘recommending opportunities for training when this is appropriate’, with only 60.3% of supervisors in the baseline sample who usually or always do this and 23.5% who never/rarely do this. The end-line sample was more positive, with 72.8% responding that they usually or always recommend opportunities for training where appropriate. At baseline, 11.8% of supervisors admitted that they never or rarely ‘provide constructive negative feedback to staff if necessary’, with 72.1% of supervisors responding that they usually or always do this. Results were more positive in the end-line sample, with only 3.7% of supervisors responding that they never or rarely carry out this activity, and 88.9% responding that they usually or always do this. Total scores on ‘maintaining high levels of performance’ for baseline and end-line samples (Table 12 and 14) show that in the control group, scores for the end-line sample are in fact slightly lower than for the baseline sample. In both intervention groups, the end-line samples have generally higher scores than the corresponding baseline samples. However, the difference in this measure between baseline and end-line is only statistically significant in intervention a + b (*p* < 0.05).

**Table 5:** Supervisors (a + b and a + b + c) “To maintain high levels of performance…”

Item	Baseline (N = 68)			End-line (N = 81)		
	never/rarely	some-times	usually/always	never/rarely	some-times	usually/always
I jointly develop work objectives with employees	5 (7.4%)	11 (16.2%)	52 (76.5%)	2 (2.5%)	11 (13.6%)	68 (84%)
I agree on performance standards with the employee	4 (5.9%)	18 (26.5%)	46 (67.6%)	6 (7.4%)	11 (13.6%)	64 (79%)
I give employees adequate information on how well they are performing	4 (5.9%)	11 (16.2%)	53 (77.9%)	2 (2.5%)	7 (8.6%)	72 (88.9%)
I publicly acknowledge individual accomplishments	3 (4.4%)	7 (10.3%)	58 (85.3%)	2 (2.5%)	5 (6.2%)	74 (91.4%)
I take staff ideas, suggestions and wishes into account whenever possible	3 (4.4%)	8 (11.8%)	57 (83.8%)	1 (1.2%)	5 (6.2%)	75 (92.6%)
I recommend opportunities for training when this is appropriate	16 (23.5%)	11 (16.2%)	41 (60.3%)	12 (14.8%)	10 (12.3%)	59 (72.8%)
I provide positive and constructive feedback,to staff	3 (4.4%)	5 (7.4%)	60 (88.2%)	0 (0%)	8 (9.9%)	73 (90.1%)
I provide constructive negative feedback to staff if necessary	8 (11.8%)	11 (16.2%)	49 (72.1%)	3 (3.7%)	6 (7.4%)	72 (88.9%)
I use fair and objective standards to evaluate staff performance	5 (7.4%)	4 (5.9%)	59 (86.8%)	2 (2.5%)	6 (7.4%)	73 (90.1%)

Health workers were asked about activities carried out by their supervisors to maintain high levels of performance. The responses are summarized in Table 6 for intervention group (a + b and a + b + c). At baseline, 26.1% of health workers responded that their supervisor never or rarely ‘recommends opportunities for training...’, with less than one third responding that their supervisor usually or always did this. The end-line sample presents a different picture, with just 8.1% of intervention a + b and a + b + c health workers specifying that their supervisor never/rarely recommends opportunities for training where appropriate, and 81.9% responding that their supervisor usually or always does this. This difference between the baseline and end-line samples reflects the differences in the supervisors’ own attitudes. Furthermore, at baseline, less than two thirds (64.7%) of sampled health workers (intervention group a + b and a + b + c) responded that their supervisor usually or always ‘uses fair measures and guidelines to assess how well I’m doing’. At end-line, this percentage increased to 86.6% of health workers. The mean total score on ‘maintaining high levels of performance’ was not significantly different between baseline and end-line for the control group, but was significantly different (with higher mean scores at end-line) for intervention groups a + b and a + b + c, reflecting the trend observed in the supervisor data.

**Table 6:** Health Workers (a + b and a + b + c) “To maintain high levels of performance, my supervisor…”

Item	Baseline (N = 153)			End-line (N = 149)		
	never/rarely	some-times	usually/always	never/rarely	some-times	usually/always
Works with me to develop clear learning targets....	15 (9.8%)	25 (16.3%)	113 (73.9%)	2 (1.3%)	26 (17.4%)	121 (81.2%)
Agrees with me the standard of my performance…	9 (5.9%)	22 (14.4%)	122 (79.7%)	2 (1.3%)	12 (8.1%)	135 (90.6%)
Gives me enough information on how well I'm performing	31 (20.3%)	17 (11.1%)	105 (68.6%)	10 (6.7%)	16 (10.7%)	123 (82.6%)
Publicly acknowledges my accomplishments	24 (15.7%)	22 (14.4%)	107 (69.9%)	10 (6.7%)	17 (11.4%)	122 (81.9%)
Takes my ideas, suggestions and wishes into account whenever possible	20 (13.1%)	15 (9.8%)	118 (77.1%)	6 (4%)	16 (10.7%)	127 (85.2%)
Recommends opportunities for training…	40 (26.1%)	14 (9.2%)	99 (64.7%)	12 (8.1%)	15 (10.1%)	122 (81.9%)
Gives me helpful feedback on the things I do well	24 (15.7%)	27 (17.6%)	102 (66.7%)	10 (6.7%)	15 (10.1%)	124 (83.2%)
Gives me helpful feedback on the things I need to do better	23 (15%)	26 (17%)	104 (68%)	10 (6.7%)	11 (7.4%)	128 (85.9%)
Uses fair measures and guidelines to assess how well I'm doing	33 (21.6%)	21 (13.7%)	99 (64.7%)	9 (6%)	11 (7.4%)	129 (86.6%)

Supervisors in interventions a + b and a + b + c had higher scores in general on ‘dealing with performance problems’ at end-line than at baseline Table 7. Specifically, at baseline, close to 20% of supervisors responded that they never, rarely or sometimes ‘assess whether additional training may be needed for skill deficiency’. At end-line, this percentage was much lower (6.2% of end-line supervisors). Tables 12 and 14 show that in both intervention groups, the total scores on ‘dealing with performance problems’ are higher on average at end-line than at baseline though this difference is more marked in intervention a + b (*p* < 0.05). In contrast, mean total score on ‘dealing with performance problems’ in the control group was slightly lower at end-line than at baseline. At end-line, the percentage of supervisors who responded that they usually or always ‘listen, guide and encourage the employee to solve his/her own problems is 93.8%, quite a bit higher than the corresponding baseline percentage of 77.9%.

**Table 7:** Supervisors (a + b and a + b + c) “When dealing with performance problems…”

Item	Baseline (N = 68)			End-line (N = 81)		
	never/rarely	some-times	usually/always	never/rarely	some-times	usually/always
I look for possible causes and describe the problem objectively	5 (7.4%)	9 (13.2%)	54 (79.4%)	3 (3.7%)	5 (6.2%)	73 (90.1%)
I don’t assign blame for the problem without having a full understanding of the problem	6 (8.8%)	2 (2.9%)	60 (88.2%)	1 (1.2%)	2 (2.5%)	78 (96.3%)
I assess whether additional training may be needed for skill deficiency	2 (2.9%)	11 (16.2%)	55 (80.9%)	3 (3.7%)	2 (2.5%)	76 (93.8%)
I establish a joint action plan with the employee(s) to solve the problem	7 (10.3%)	7 (10.3%)	54 (79.4%)	2 (2.5%)	7 (8.6%)	72 (88.9%)

At end-line, health workers in intervention groups a + b and a + b + c were very positive overall about how their supervisors deal with performance problems (Table 8). For example, a large majority (87.2%) felt that their supervisors usually or always ‘[don’t] blame anyone unless they are really sure about the problem’—compared with 67.3% of the baseline health workers in these intervention groups. Trends in total scores on ‘dealing with performance problems’ are reflective of those of supervisors, with increases in the mean total score in intervention groups a + b and a + b + c (*p* < 0.05), and a slight decrease at end-line in total score on this section for the control group.

**Table 8:** Health Workers (a + b and a + b + c) “When dealing with performance problems, my supervisor…”

Item	Baseline (N = 153)			End-line (N = 149)		
	never/rarely	sometimes	usually/always	never/rarely	sometimes	usually/always
Tries to find out what caused the problem and describes the problem fairly	25 (16.3%)	24 (15.7%)	104 (68%)	6 (4%)	15 (10.1%)	128 (85.9%)
Doesn't blame anyone unless they are really sure about the problem	28 (18.3%)	22 (14.4%)	103 (67.3%)	9 (6%)	10 (6.7%)	130 (87.2%)
Sees if training to give staff more skills would help to fix the problem	22 (14.4%)	23 (15%)	108 (70.6%)	10 (6.7%)	13 (8.7%)	126 (84.6%)
Works with staff to plan how to fix the problem	20 (13.1%)	14 (9.2%)	119 (77.8%)	6 (4%)	9 (6%)	134 (89.9%)

In the supervisors survey, total scores on ‘counselling a troubled employee’ are higher in general for the end-line samples than the corresponding baseline samples for intervention groups a + b and a + b + c (*p* < 0.05), and particularly for intervention a + b + c. For the control group, the opposite is the case, with lower mean scores in general at end-line than at baseline (Table 12 and 14). Again, trends in health worker responses matched those of supervisor responses.

When asked about how their supervisor deals with problems, the end-line sample of health workers (intervention a + b and a + b + c districts) were more positive than the baseline sample about how their supervisors deal with their (health workers’) problems. At baseline, less than two thirds (65.4%) of the sample responded that their supervisor usually or always ‘puts me in touch with a service that can help me, if this is what I need’, while at end-line this percentage was much higher at 84.6% (Table 9 and 10). Total scores on this section were higher on average at end-line than at baseline for health workers in both intervention groups (*p* < 0.05) but were slightly lower on average at end-line than at baseline for the control group, as with the supervisor survey.

**Table 9:** Supervisors (a + b and a + b + c) “When counselling a troubled employee …”

Item	Baseline (N = 68)			End-line (N = 81)		
	never/rarely	some-times	usually/always	never/rarely	some-times	usually/always
I offer assistance when known problems or difficulties interfere with staff job performance	2 (2.9%)	4 (5.9%)	62 (91.2%)	0 (0%)	1 (1.2%)	80 (98.8%)
I listen, guide and encourage the employee to solve his/her own problems	7 (10.3%)	8 (11.8%)	53 (77.9%)	1 (1.2%)	4 (4.9%)	76 (93.8%)
I refer them to an appropriate support service if necessary	4 (5.9%)	5 (7.4%)	59 (86.8%)	1 (1.2%)	3 (3.7%)	77 (95.1%)

**Table 10:** Health Workers (a + b and a + b + c) “If I have problems, my supervisor…”

Item	Baseline (N = 153)			End-line (N = 149)		
	never/rarely	some-times	usually/always	never/rarely	some-times	usually/always
Helps me if the problem is affecting how well I do my job	17 (11.1%)	22 (14.4%)	114 (74.5%)	9 (6%)	12 (8.1%)	128 (85.9%)
Listens to me and encourages me to solve my problem	20 (13.1%)	14 (9.2%)	119 (77.8%)	7 (4.7%)	8 (5.4%)	134 (89.9%)
Puts me in touch with a service that can help me, if this is what I need	34 (22.2%)	19 (12.4%)	100 (65.4%)	7 (4.7%)	16 (10.7%)	126 (84.6%)

Supervisors were asked about three activities that they carry out to manage their time (Table 11). A larger percentage (91.4%) of the end-line supervisor sample reported that they usually/always ‘plan [their] daily, weekly and monthly schedule to allow time for the most important tasks’, than in the baseline sample (77.9% of baseline supervisors). Total scores on time management were generally higher at end-line than at baseline for supervisors in intervention a + b, with little difference between baseline and end-line scores for supervisors in the control and intervention a + b + c districts). However, intervention a + b + c scored generally highly on time management at baseline, with at least 50% of supervisors getting the maximum possible score of 15, and therefore, there was not so much scope for improvement as in intervention a + b.

**Table 11:** Supervisor (a + b and a + b + c) time management…

Item	Baseline (N = 68)			End-line (N = 81)		
	never/rarely	some-times	usually/always	never/rarely	some-times	usually/always
I plan my daily, weekly and monthly schedule to allow time for the most important tasks…	5 (7.4%)	10 (14.7%)	53 (77.9%)	3 (3.7%)	4 (4.9%)	74 (91.4%)
I delegate tasks wherever possible	1 (1.5%)	2 (2.9%)	65 (95.6%)	1 (1.2%)	4 (4.9%)	76 (93.8%)
I ask my manager for advice when I have too much work and not enough time to carry out the supervision	3 (4.4%)	5 (7.4%)	60 (88.2%)	4 (4.9%)	2 (2.5%)	75 (92.6%)

Overall, there are some noticeable differences in the baseline and end-line scores for supervisors in interventions a + b and a + b + c. Table 12 shows the difference in mean (with standard error) and median scores on CSAI between baseline and end-line samples, by intervention group. Also shown are the *p* values from Mann-Whitney *U* tests for a difference in the distribution of total scores between baseline and end-line (Table 13). The rightmost column in the table shows the *p* values adjusted for multiple testing error (FDR-p). For the control group, there is little difference in the mean and median scores from baseline to end-line, with end-line mean scores slightly lower than the baseline mean scores. This is also reflected in the lack of significant differences between baseline and end-line scores. For intervention a + b, significant differences between baseline and end-line were observed in the total scores on ‘maintaining high levels of performance’, ‘dealing with performance problems’, ‘counselling a troubled employee’ and ‘time management’. In contrast, for intervention a + b + c, a significant difference in distribution of scores was only found on ‘counselling a troubled employee’, although the end-line mean scores were higher than their corresponding baseline mean scores for all parts of the CSAI. The fewer significant differences for intervention a + b + c could possibly be explained by the fact that the baseline scores in this group tended to be higher to begin with, leaving less scope for improvement.

**Table 12:** Supervisors – summary of total scores by intervention group

Total score	Stage of data collection	INTERVENTION GROUP								
		Control			a + b			a + b + c		
		Mean	SD	Median	Mean	SD	Median	Mean	SD	Median
In my interactions with staff	Baseline	21.56	2.12	21	21.81	2.52	22	22.19	2.79	23
	End-line	21.3	2.58	21	22.4	2.62	23	22.8	2.23	23
To maintain high levels of performance	Baseline	37.37	3.7	37	35.34	4.53	35	37.83	6.69	39
	End-line	36.44	5.22	36	38.83	5.28	39	39.59	5.44	42
When dealing with performance problems	Baseline	17.26	2.18	17	16.31	2.56	16	17.22	3.4	18
	End-line	16.59	2.87	16	18.3	1.83	19	17.9	2.52	19
Counselling a troubled employee	Baseline	13.11	1.72	13	12.91	1.86	13	12.89	2.35	13.5
	End-line	12.07	2.62	12	13.9	1.24	14	14	1.48	15
Time management	Baseline	12.63	2.1	13	12.75	2.05	13	13.5	2.08	15
	End-line	12.52	2.23	13	13.9	1.39	14	13.61	2.12	15

**Table 13:** Comparison of total scores at baseline and end-line (Supervisors)

Total Score	Intervention group	Difference in sample means (end-line - baseline)	Standard error of the difference in sample means	Difference in sample medians (end-line - baseline)	Mann-Whitney U test
In my interactions with staff	Control	-0.26	0.64	0	U = 344, Z = -0.36, p-value =0.72
	a + b	0.59	0.61	1	U = 524, Z = -1.33, p-value = 0.184
	a + b + c	0.61	0.58	0	U = 661, Z = -0.8, p-value = 0.424
To maintain high levels of performance	Control	-0.93	1.23	-1	U = 331, Z = -0.58, p-value =0.56
	a + b	3.48	1.16	4	U = 373, Z = -3.03, **p-value = 0.002**
	a + b + c	1.75	1.40	3	U = 624, Z = -1.17, p-value = 0.243
When dealing with performance problems	Control	-0.67	0.69	-1	U = 310, Z = -0.95, p-value =0.34
	a + b	1.99	0.54	3	U = 347.5, Z = -3.38, **p-value = 0.001**
	a + b + c	0.68	0.69	1	U = 669.5, Z = -0.72, p-value = 0.473
Counselling a troubled employee	Control	-1.04	0.60	-1	U = 285, Z = -1.41, p-value =0.159
	a + b	0.99	0.38	1	U = 433.5, Z = -2.43, **p-value = 0.015**
	a + b + c	1.11	0.46	1.5	U = 511.5, Z = -2.42, **p-value = 0.015**
Time management	Control	-0.11	0.59	0	U = 356.5, Z = -0.14, p-value =0.888
	a + b	1.15	0.42	1	U = 425.5, Z = -2.52, **p-value = 0.012**
	a + b + c	0.11	0.48	0	U = 721, Z = -0.19, p-value = 0.85

Total scores were calculated for each part of CSAI on the health worker survey, by summing over items. Table 14 shows the means, medians and standard deviations of these total scores for each intervention group at baseline and end-line. Table 15 shows the difference in the mean and median values between baseline and end-line for each subscore, the standard error of the difference in means, and results of a Mann-Whitney *U* test to test for differences in the distribution of total scores between the baseline and end-line samples. In both intervention groups, the mean subscores were higher for the end-line samples than the baseline, across all parts of section C. The differences in sample means were larger in intervention group a + b + c than in intervention group a + b for all subscores, except the last, ‘If I have problems, my supervisor…’. For intervention group a + b + c, significant differences between baseline and end-line in the distribution of total scores were observed for ‘How my supervisor interacts with staff’, ‘How my supervisor maintains high levels of performance’ and ‘How my supervisor deals with performance problems’. For intervention group a + b, significant differences in distribution between baseline and end-line samples were observed for ‘How my supervisor maintains high levels of performance’ and for ‘If I have problems, my supervisor…’.

**Table 14:** Health Workers - Summary of total scores by intervention group

Total Score	Stage of data collection	Control			Intervention A + B			Intervention A + B + C		
		Mean	SD	Median	Mean	SD	Median	Mean	SD	Median
How my supervisor interacts with staff -	Baseline	20.51	4.6	22	20.61	3.86	21	20.6	4.5	22
	End-line	19.74	4.02	20	21.52	3.06	21	22.16	3.32	23
How my supervisor maintains high levels of performance	Baseline	34.98	8.79	37	34.81	8.13	36	35.38	8.22	37
	End-line	34.29	7.22	34	37.93	6.85	39	38.72	5.94	39
How my supervisor deals with performance problems - sum of items	Baseline	15.93	4.58	17	15.98	3.72	17	15.62	3.84	16
	End-line	15.61	3.25	16	17.22	2.87	18	17.51	3.15	19
If I have problems, my supervisor… -	Baseline	12.09	3.42	13	11.63	3.21	12	12.18	3.32	13
	End-line	11.61	2.91	12	12.74	2.64	13	13.16	2.14	14

**Table 15:** Change from baseline to end-line in total scores (Health Workers)

Total scores	Intervention group	Difference in sample means (end-line - baseline)	Standard error of the difference in sample means	Difference in sample medians (end-line - baseline)	Mann-Whitney U test
How my supervisor interacts with staff	Control	-0.15	0.19	-0.4	U = 687, Z = -1.237, p-value = 0.216
	a + b	0.18	0.12	0	U = 1877, Z = -1.219, p-value =0.223
	a + b + c	0.31	0.12	0.2	U = 2963.5, Z = -2.126, **p-value = 0.033**
How my supervisor maintains high levels of performance	Control	-0.08	0.2	-0.33	U = 731.5, Z = -0.811, p-value = 0.418
	a + b	0.34	0.15	0.33	U = 1663, Z = -2.206, **p-value =0.027**
	a + b + c	0.37	0.12	0.22	U = 2809, Z = -2.586, **p-value = 0.01**
How my supervisor deals with performance problems	Control	-0.08	0.22	-0.25	U = 685, Z = -1.262, p-value = 0.207
	a + b	0.3	0.15	0.25	U = 1757.5, Z = -1.788, p-value =0.074
	a + b + c	0.48	0.13	0.75	U = 2373, Z = -3.976, **p-value = <0.001**
If I have problems, my supervisor…	Control	-0.16	0.23	-0.33	U = 670, Z = -1.409, p-value = 0.159
	a + b	0.37	0.17	0.33	U = 1703, Z = -2.06, **p-value =0.039**
	a + b + c	0.33	0.14	0.34	U = 3180.5, Z = -1.474, p-value = 0.14

Thus, from a health worker’s perspective, STEM may have had a larger impact in intervention group a + b + c on how supervisors interact with staff, how supervisor maintain high levels of performance and on how supervisors handle health workers’ problems.

### Changes on some supervisory roles after STEM

At end-line, supervisors in intervention a + b and a + b + c districts were asked general questions about some aspects of their jobs that may have changed since the STEM training; their responses are summarized in Table 16.

**Table 16:** Some changes outlined as a result of the STEM intervention (a+b and a+b+c)

Item	much worse	a little worse	about the same	a little better	much better	missing
The amount of preparation required for your job	1 (2.3%)	0 (0%)	1 (2.3%)	8 (18.6%)	33 (76.7%)	38
Communication with D/CHMT	1 (2.4%)	0 (0%)	2 (4.8%)	9 (21.4%)	30 (71.4%)	39
Relationships with D/CHMT	0 (0%)	0 (0%)	1 (2.4%)	10 (23.8%)	31 (73.8%)	39
Your overall workload	2 (4.8%)	2 (4.8%)	4 (9.5%)	17 (40.5%)	17 (40.5%)	39
Problem-solving with D/CHMT	0 (0%)	1 (2.3%)	3 (7.0%)	18 (41.9%)	21 (48.8%)	38
Problem-solving within the facility	0 (0%)	1 (2.4%)	1 (2.4%)	6 (14.3%)	34 (81%)	39
Your overall level of job satisfaction	0 (0%)	0 (0%)	1 (2.4%)	12 (28.6%)	29 (69%)	39
Your personal motivation	3 (7.1%)	1 (2.4%)	4 (9.5%)	11 (26.2%)	23 (54.8%)	39
Treating staff with respect and recognizing their contribution	0 (0%)	0 (0%)	1 (2.4%)	4 (9.8%)	36 (87.8%)	40
Using fair procedures to make decisions	0 (0%)	0 (0%)	1 (2.3%)	8 (18.2%)	35 (79.5%)	37
Communicating ethical expectations - organizational and professional	1 (2.4%)	1 (2.4%)	2 (4.9%)	10 (24.4%)	27 (65.9%)	40
Holding staff, including yourself, accountable	0 (0%)	0 (0%)	1 (2.3%)	14 (32.6%)	28 (65.1%)	38
Rewarding and supporting employees who behave ethically	1 (2.3%)	1 (2.3%)	2 (4.7%)	4 (9.3%)	35 (81.4%)	38

About half of the supervisors did not respond to these questions, so it is likely that these did not complete the STEM training. Of the supervisors who completed this section, responses were very positive. A large majority of supervisors (87.8%) felt that they were much better at ‘treating staff with respect and recognizing their contribution’, with a further 9.8% responding that they were ‘a little better’ at this. Furthermore, 81% of the supervisors felt that ‘problem solving within the facility’ was much better, while 14.3% felt it was a little better. The least improvement was seen regarding ‘overall workload’, with 81% saying this was either a little or much better, 9.5% about the same, and 9.5% (4 supervisors) a little worse or much worse.

## Discussion

The results of the study suggest that the intervention package, which includes workshops, 1-week intensive training and 12 months of action learning sets, within 18 months can contribute to improved understanding and application of supportive supervision by supervisors and help remove some self-perceived barriers such as time management and lack of confidence. The absence of supervision or the provision of poor quality, sporadic supervision has a demotivating effect on frontline health workers. A key aim of this intervention was to improve the working environment and specifically supervision, by targeting managers and their approach to HRM. Improvements in working environment were determined through changes in the perceptions and experiences of supervisors and health workers in the facilities selected for participation in this study. The results indicated an improvement in the intervention a + b and a + b + c districts. In both intervention groups, the end-line samples have generally higher scores than the corresponding baseline samples for both supervisors and health workers. However, the difference is more marked in intervention a + b for the supervisors and in intervention a + b + c for health workers. This provides evidence of the positive impact of the intervention on supervisors’ behaviours in the intervention groups, compared with the control group and demonstrates that supervisors are making procedural changes within their facilities which will in turn have a positive impact on staff. Of concern was that only half of the supervisors and health workers who participated in the baseline survey were available and participated at the end-line survey suggesting that there is a substantial amount of transfers and movement of health workers between facilities and districts. This may have some implications on human resource for health planning especially as it relates to human resource management strategies in district health systems because of the lack of continuity.

The study has several limitations. First, the unavailability of many of the baseline sample at end-line has reduced the power of the study. The fact that the pre- and post-intervention surveys were completed by different individuals in many cases (i.e. the data was not paired) meant that it was not possible to calculate individual *changes in scores* and compare *change in scores* between intervention groups, and between control and intervention. Furthermore, the study was powered to detect only a medium-large effect size for the supervisor sample; the study is not sufficiently powered to detect small effect sizes for most analyses.

Our measure on supportive supervision was based on supervisor’s self- reported perceived performance and the health workers’ perceptions of their supervisors’ performance at baseline and end-line. This may not be a complete reflection of the support provided by the supervisors and/or received by the health workers.

In our previous studies and also from previous research, we have observed that motivation is difficult to maintain in a supervisory culture of inspection and fault finding using a checklist approach [2, 3, 17, 18]. STEM is one of the few interventions that has attempted to address this problem in sub-Saharan Africa and, despite its limitations, interventions like STEM can be used to support existing policy. With some countries like Tanzania moving towards a supportive supervision approach and the government publication of National Supportive Supervision Guidelines to address earlier shortcomings in human resource management [19], a systematic approach to supportive supervision is recognised as a vital component of HRM and critical to development of the health workforce. In an earlier research study in Tanzania exploring districts health managers’ perception of supervision, policy-level attention to the importance of supportive supervision as a tool for advancing health sector objectives was observed and the managers’ attitudes suggested a paradigm of teaching, problem solving and improvement. This reflects a national commitment, reinforced with clear mechanisms, structures and shared expectations that views supportive supervision and the attitudes upon which this is based as a necessary part of the HRM process [20]. Supervisors felt that STEM provided them the skill set to be a good supervisor and the support to further develop these skills. The benefits of developing such skill sets have been documented in literature and they include increased job satisfaction, improved health worker motivation, better ability to identify problems and solve them in a timely manner, identifying staff needs and providing opportunities for personal development [17, 21–23].

Lack of quality human resource management can affect health care systems in Tanzania and our result shows a general improvement of supervisor’s knowledge and practice of supportive supervision as a result of training in supportive supervision and the use of action learning as a team work and problem solving tool within the facilities. Thus training in supportive supervision using interventions such as STEM may be helpful not only for improving supervisors knowledge but also to promote the acceptance and practice of supportive supervision and positively influence health workers motivation and satisfaction with their supervision. The movement of staff within and across facilities and districts may prove a challenge to such an approach but this can be addressed by policy interventions to reduce health workers transfer outside their districts.

Though the analysis demonstrated several benefits of STEM on supervisors’ perceptions of their competencies, there are several factors which can affect the success of supportive supervision beyond the capacity of supervisors. This may include the organisational structure and the health care system. Logistical and financial constraints are major limitations to supportive supervision in low-income countries.

## Conclusion

In conclusion, the intervention has developed, training materials and learning tools to enable effective supportive supervision to happen at a facility level. In the districts where this has been implemented, there is evidence of increased supervisory activity at the facility level, improved supervision competency, and increased health worker satisfaction with supervision. The government of Tanzania should further explore the success of the intervention by continuing the process in the districts studied and scaling up the implementation of the model in other districts for sustainable results. The materials and tools for the intervention have been made available to the Ministry and civil society organisations, and we suggest that a plan be put in place to extend the intervention beginning with the control districts in this study. We strongly advise continued monitoring of work environment indicators and retention rates to track whether the positive outcomes from the intervention endure over time.

## Additional files

Additional file 1: STEM health facilities, by region, district and intervention group. (DOCX 48 bytes)

Additional file 2: Reliability statistics (Cronbach's alpha) for the adapted Supervisor Competency Self-Assessment Inventory (CSAI) in the health worker survey. (DOCX 13 bytes)

Additional file 3: Reliability statistics (Cronbach's alpha) for the adapted Supervisor Competency Self-Assessment Inventory (CSAI) in the supervisor survey. (DOCX 13 bytes)
