# Supplementary material for: The impact of a human resource management intervention on the capacity of supervisors to support and supervise their staff at health facility level
Source: Hum Resour Health. 2017 Aug 30;15:57. doi: 10.1186/s12960-017-0225-0 (PMC5577784; doi:10.1186/s12960-017-0225-0)
Supplement: Supplementary file 1 — STEM health facilities, by region, district and intervention group. (DOCX 48 bytes) [file 12960_2017_225_MOESM1_ESM.docx]

Table Supplementary 1: STEM health facilities, by region, district and intervention group

|  | Intervention group | | |
| --- | --- | --- | --- |
|  | control | a+b | a+b+c |
| Region | Tanga | | |
| District | Handeni | Muheza | Tanga Urban |
| Facility | Kabuku Hc | Mkanyageni Disp | Duga Disp |
|  | Kideleko Hc | Mkuzi Hc | Kisosora Hc |
|  | Konje Disp | Ngarani Kwakifua Disp | Mafuriko Disp |
|  | Misima Dis | Ngomeni Umba Disp | Ngamiani Hc |
|  | Mkata Hc | Ubwari Disp | Pande Disp |
|  | | | |
| Region | Iringa | | |
| District | Ludewa | Mufindi | Iringa Urban |
| Facility | Lupanga Disp | Ihongole Hc | Igumbilo Disp |
|  | Madilu Disp | Kasanga Hc | Ipogolo Hc |
|  | Manda Hc | Malangali Hc | Kitwiru Disp |
|  | Mawengi Disp | Mgololo Hc | Ngome Hc |
|  | Mlangali Hc | Sadani Hc | Sabasaba Disp |
|  | | | |
| Region | Tabora | | |
| District | Urambo | Nzega | Tabora Urban |
| Facility | Magereza Disp | Bukene Hc | Cheyo Disp |
|  | Songambele Disp | Busondo Hc | Isevya Disp |
|  | Ulyankulu Hc | Itobo Hc | Kiloleni Disp |
|  | Usisya Disp | Nata Disp | Ng?ambo Disp |
|  | Usoke Hc | Zogolo Hc | Town Disp |

Table 1 Demographics of Supervisors

| **Stage** | **Baseline** | | | **Endline** | | |
| --- | --- | --- | --- | --- | --- | --- |
| **Intervention group** | **Control** | **A+B** | **A+B+C** | **Control** | **A+B** | **A+B+C** |
| Number of supervisors | 27 | 32 | 36 | 27 | 40 | 41 |
| ***Gender*** | | | | | | |
| Male | 12 (44.4%) | 10 (31.2%) | 11 (30.6%) | 12 (44.4%) | 10 (25%) | 12 (29.3%) |
| Female | 15 (55.6%) | 22 (68.8%) | 25 (69.4%) | 15 (55.6%) | 30 (75%) | 29 (70.7%) |
| *Age in years* | | | | | | |
| Mean (sd) | 46.9 (8.7) | 43.9 (10.5) | 41.1 (8.5) | 40.4 (11.4) | 41.4 (10.8) | 40.7 (10.1) |
| ***Highest medical or paramedical qualification*** | | | | | | |
| Medical Officer | 2 (7.4%) | 0 (0%) | 1 (2.8%) | 2 (7.4%) | 1 (2.5%) | 1 (2.4%) |
| Assistant Medical Officer | 1 (3.7%) | 4 (12.5%) | 3 (8.3%) | 1 (3.7%) | 4 (10%) | 5 (12.2%) |
| Clinical officer | 9 (33.3%) | 7 (21.9%) | 12 (33.3%) | 4 (14.8%) | 10 (25%) | 9 (22%) |
| Assistant clinical officer | 1 (3.7%) | 1 (3.1%) | 2 (5.6%) | 3 (11.1%) | 2 (5%) | 3 (7.3%) |
| Medical attendant | 2 (7.4%) | 1 (3.1%) | 1 (2.8%) | 3 (11.1%) | 0 (0%) | 2 (4.9%) |
| Registered nurse | 3 (11.1%) | 6 (18.8%) | 12 (33.3%) | 7 (25.9%) | 11 (27.5%) | 12 (29.3%) |
| Registered nurse midwife | 3 (11.1%) | 7 (21.9%) | 3 (8.3%) | 3 (11.1%) | 7 (17.5%) | 5 (12.2%) |
| Enrolled nurse | 2 (7.4%) | 2 (6.2%) | 1 (2.8%) | 0 (0%) | 3 (7.5%) | 3 (7.3%) |
| Enrolled nurse midwife | 3 (11.1%) | 3 (9.4%) | 1 (2.8%) | 2 (7.4%) | 0 (0%) | 0 (0%) |
| MCH Aides | 1 (3.7%) | 1 (3.1%) | 0 (0%) | 2 (7.4%) | 2 (5%) | 1 (2.4%) |
| ***Current job position*** | | | | | | |
| Facility incharge | 12 (44.4%) | 11 (34.4%) | 15 (41.7%) | 8 (29.6%) | 12 (30%) | 15 (36.6%) |
| Assistant facility incharge | 1 (3.7%) | 1 (3.1%) | 2 (5.6%) | 3 (11.1%) | 4 (10%) | 5 (12.2%) |
| Head of unit | 10 (37%) | 14 (43.8%) | 13 (36.1%) | 10 (37%) | 14 (35%) | 16 (39%) |
| Head of department | 4 (14.8%) | 4 (12.5%) | 6 (16.7%) | 1 (3.7%) | 2 (5%) | 0 (0%) |
| Other | 0 (0%) | 2 (6.2%) | 0 (0%) | 5 (18.5%) | 8 (20%) | 5 (12.2%) |

**Table 2 Demographics of Health workers**

| **Stage** | **Baseline** | | | **Endline** | | |
| --- | --- | --- | --- | --- | --- | --- |
| **Intervention group** | **Control** | **A+B** | **A+B+C** | **Control** | **A+B** | **A+B+C** |
| Number of health workers | 43 | 62 | 91 | 38 | 69 | 80 |
| ***Gender*** | | | | | | |
| male | 13 (30.2%) | 15 (24.6%) | 13 (14.3%) | 12 (31.6%) | 13 (19.1%) | 9 (11.2%) |
| female | 30 (69.8%) | 46 (75.4%) | 78 (85.7%) | 26 (68.4%) | 55 (80.9%) | 71 (88.8%) |
| ***Age in years*** | | | | | | |
| Mean (sd) | 44 (10) | 40 (10) | 39 (10) | 36 (10) | 37 (11) | 36 (11) |
| ***Highest medical or paramedical qualification*** | | | | | | |
| Medical Officer | 0 (0%) | 0 (0%) | 0 (0%) | 0 (0%) | 0 (0%) | 1 (1.2%) |
| Assistant Medical Officer | 1 (2.3%) | 0 (0%) | 3 (3.3%) | 0 (0%) | 0 (0%) | 1 (1.2%) |
| Clinical officer | 3 (7%) | 7 (11.3%) | 1 (1.1%) | 0 (0%) | 3 (4.3%) | 0 (0%) |
| Assistant clinical officer | 0 (0%) | 3 (4.8%) | 6 (6.6%) | 0 (0%) | 4 (5.8%) | 3 (3.8%) |
| Medical attendant | 22 (51.2%) | 34 (54.8%) | 42 (46.2%) | 19 (50%) | 21 (30.4%) | 35 (43.8%) |
| Registered nurse | 5 (11.6%) | 4 (6.5%) | 17 (18.7%) | 10 (26.3%) | 20 (29%) | 17 (21.2%) |
| Registered nurse midwife | 3 (7%) | 6 (9.7%) | 7 (7.7%) | 3 (7.9%) | 11 (15.9%) | 11 (13.8%) |
| Enrolled nurse | 3 (7%) | 3 (4.8%) | 9 (9.9%) | 2 (5.3%) | 2 (2.9%) | 5 (6.2%) |
| Enrolled nurse midwife | 4 (9.3%) | 1 (1.6%) | 4 (4.4%) | 1 (2.6%) | 5 (7.2%) | 3 (3.8%) |
| MCH Aides | 1 (2.3%) | 1 (1.6%) | 1 (1.1%) | 3 (7.9%) | 3 (4.3%) | 4 (5%) |
| Laboratory staff | 1 (2.3%) | 3 (4.8%) | 1 (1.1%) | 0 (0%) | 0 (0%) | 0 (0%) |
| ***Number of years working at health facility*** | | | | | | |
| Mean (sd) | 8.8 (8.9) | 8.7 (9) | 2.9 (2.6) | 4 (4.5) | 7.6 (7.4) | 3.5 (2.7) |
| ***Current job position*** |  |  |  |  |  |  |
| facility incharge | 0 (0%) | 0 (0%) | 0 (0%) | 0 (0%) | 1 (1.4%) | 1 (1.2%) |
| assistant facility incharge | 2 (4.7%) | 3 (4.8%) | 3 (3.3%) | 1 (2.6%) | 2 (2.9%) | 2 (2.5%) |
| head of unit | 0 (0%) | 0 (0%) | 0 (0%) | 1 (2.6%) | 2 (2.9%) | 2 (2.5%) |
| head of department | 1 (2.3%) | 3 (4.8%) | 2 (2.2%) | 0 (0%) | 1 (1.4%) | 0 (0%) |
| other | 40 (93%) | 56 (90.3%) | 86 (94.5%) | 36 (94.7%) | 63 (91.3%) | 75 (93.8%) |

Table 3A Supervisors (intervention a+b and a+b+c) - In interactions with staff…

| **Item** | **Baseline (N=68)** | | | **End-line (N=81)** | | |
| --- | --- | --- | --- | --- | --- | --- |
|  | **never/ rarely** | **some-times** | **usually/ always** | **never/ rarely** | **some-times** | **usually/ always** |
| I communicate my general expectations about performance to staff | 5 (7.4%) | 4 (5.9%) | 59 (86.8%) | 4 (4.9%) | 7 (8.6%) | 70 (86.4%) |
| I listen to staff and am open to their concerns | 3 (4.4%) | 6 (8.8%) | 59 (86.8%) | 0 (0%) | 7 (8.6%) | 74 (91.4%) |
| I actively attempt to solve problems in the health facility | 5 (7.4%) | 18 (26.5%) | 45 (66.2%) | 4 (4.9%) | 12 (14.8%) | 65 (80.2%) |
| I treat people fairly and consistently | 0 (0%) | 3 (4.4%) | 65 (95.6%) | 0 (0%) | 1 (1.2%) | 80 (98.8%) |
| I respect staff and their contributions | 0 (0%) | 2 (2.9%) | 66 (97.1%) | 1 (1.2%) | 0 (0%) | 80 (98.8%) |

**Table 3B Health Workers (a+b and a+b+c) “In interactions with staff, my supervisor…”**

| **Item** | **Baseline (N=153)** | | | **End-line (N=149)** | | |
| --- | --- | --- | --- | --- | --- | --- |
|  | **never/ rarely** | **sometimes** | **usually/ always** | **never/ rarely** | **Sometimes** | **usually/ always** |
| Lets me know what is expected of me in my job. | 23 (15%) | 24 (15.7%) | 106 (69.3%) | 8 (5.4%) | 17 (11.4%) | 124 (83.2%) |
| Listens to me and takes notice of my concerns | 25 (16.3%) | 29 (19%) | 99 (64.7%) | 3 (2%) | 23 (15.4%) | 123 (82.6%) |
| Tries to take action to solve problems in the facility | 21 (13.7%) | 20 (13.1%) | 112 (73.2%) | 2 (1.3%) | 15 (10.1%) | 132 (88.6%) |
| Treats people fairly and consistently | 11 (7.2%) | 14 (9.2%) | 128 (83.7%) | 4 (2.7%) | 7 (4.7%) | 138 (92.6%) |
| Respects staff and their contributions | 11 (7.2%) | 12 (7.8%) | 130 (85%) | 2 (1.3%) | 7 (4.7%) | 140 (94%) |

**Table 4A Supervisors (a+b and a+b+c) “To maintain high levels of performance…”**

| **Item** | **Baseline (N=68)** | | | **End-line (N=81)** | | |
| --- | --- | --- | --- | --- | --- | --- |
|  | **never/ rarely** | **some-times** | **usually/ always** | **never/ rarely** | **some-times** | **usually/ always** |
| I jointly develop work objectives with employees | 5 (7.4%) | 11 (16.2%) | 52 (76.5%) | 2 (2.5%) | 11 (13.6%) | 68 (84%) |
| I agree on performance standards with the employee | 4 (5.9%) | 18 (26.5%) | 46 (67.6%) | 6 (7.4%) | 11 (13.6%) | 64 (79%) |
| I give employees adequate information on how well they are performing | 4 (5.9%) | 11 (16.2%) | 53 (77.9%) | 2 (2.5%) | 7 (8.6%) | 72 (88.9%) |
| I publicly acknowledge individual accomplishments | 3 (4.4%) | 7 (10.3%) | 58 (85.3%) | 2 (2.5%) | 5 (6.2%) | 74 (91.4%) |
| I take staff ideas, suggestions and wishes into account whenever possible | 3 (4.4%) | 8 (11.8%) | 57 (83.8%) | 1 (1.2%) | 5 (6.2%) | 75 (92.6%) |
| I recommend opportunities for training when this is appropriate | 16 (23.5%) | 11 (16.2%) | 41 (60.3%) | 12 (14.8%) | 10 (12.3%) | 59 (72.8%) |
| I provide positive and constructive feedback,to staff | 3 (4.4%) | 5 (7.4%) | 60 (88.2%) | 0 (0%) | 8 (9.9%) | 73 (90.1%) |
| I provide constructive negative feedback to staff if necessary | 8 (11.8%) | 11 (16.2%) | 49 (72.1%) | 3 (3.7%) | 6 (7.4%) | 72 (88.9%) |
| I use fair and objective standards to evaluate staff performance | 5 (7.4%) | 4 (5.9%) | 59 (86.8%) | 2 (2.5%) | 6 (7.4%) | 73 (90.1%) |

**Table 4B Health Workers (a+b and a+b+c) “To maintain high levels of performance, my supervisor…”**

| **Item** | **Baseline (N=153)** | | | **End-line (N=149)** | | |
| --- | --- | --- | --- | --- | --- | --- |
|  | **never/ rarely** | **some-times** | **usually/ always** | **never/ rarely** | **some-times** | **usually/ always** |
| Works with me to develop clear learning targets.... | 15 (9.8%) | 25 (16.3%) | 113 (73.9%) | 2 (1.3%) | 26 (17.4%) | 121 (81.2%) |
| Agrees with me the standard of my performance... | 9 (5.9%) | 22 (14.4%) | 122 (79.7%) | 2 (1.3%) | 12 (8.1%) | 135 (90.6%) |
| Gives me enough information on how well I'm performing | 31 (20.3%) | 17 (11.1%) | 105 (68.6%) | 10 (6.7%) | 16 (10.7%) | 123 (82.6%) |
| Publicly acknowledges my accomplishments | 24 (15.7%) | 22 (14.4%) | 107 (69.9%) | 10 (6.7%) | 17 (11.4%) | 122 (81.9%) |
| Takes my ideas, suggestions and wishes into account whenever possible | 20 (13.1%) | 15 (9.8%) | 118 (77.1%) | 6 (4%) | 16 (10.7%) | 127 (85.2%) |
| Recommends opportunities for training... | 40 (26.1%) | 14 (9.2%) | 99 (64.7%) | 12 (8.1%) | 15 (10.1%) | 122 (81.9%) |
| Gives me helpful feedback on the things I do well | 24 (15.7%) | 27 (17.6%) | 102 (66.7%) | 10 (6.7%) | 15 (10.1%) | 124 (83.2%) |
| Gives me helpful feedback on the things I need to do better | 23 (15%) | 26 (17%) | 104 (68%) | 10 (6.7%) | 11 (7.4%) | 128 (85.9%) |
| Uses fair measures and guidelines to assess how well I'm doing | 33 (21.6%) | 21 (13.7%) | 99 (64.7%) | 9 (6%) | 11 (7.4%) | 129 (86.6%) |

Table 5A Supervisors (a+b and a+b+c) “When dealing with performance problems…”

| **Item** | **Baseline (N=68)** | | | **End-line (N=81)** | | |
| --- | --- | --- | --- | --- | --- | --- |
|  | **never/ rarely** | **some-times** | **usually/ always** | **never/ rarely** | **some-times** | **usually/ always** |
| I look for possible causes and describe the problem objectively | 5 (7.4%) | 9 (13.2%) | 54 (79.4%) | 3 (3.7%) | 5 (6.2%) | 73 (90.1%) |
| I don’t assign blame for the problem without having a full understanding of the problem | 6 (8.8%) | 2 (2.9%) | 60 (88.2%) | 1 (1.2%) | 2 (2.5%) | 78 (96.3%) |
| I assess whether additional training may be needed for skill deficiency | 2 (2.9%) | 11 (16.2%) | 55 (80.9%) | 3 (3.7%) | 2 (2.5%) | 76 (93.8%) |
| I establish a joint action plan with the employee(s) to solve the problem | 7 (10.3%) | 7 (10.3%) | 54 (79.4%) | 2 (2.5%) | 7 (8.6%) | 72 (88.9%) |

**Table 5B Health Workers (a+b and a+b+c) “When dealing with performance problems, my supervisor…”**

| **Item** | **Baseline (N=153)** | | | **End-line (N=149)** | | |
| --- | --- | --- | --- | --- | --- | --- |
|  | **never/ rarely** | **sometimes** | **usually/ always** | **never/ rarely** | **sometimes** | **usually/ always** |
| Tries to find out what caused the problem and describes the problem fairly | 25 (16.3%) | 24 (15.7%) | 104 (68%) | 6 (4%) | 15 (10.1%) | 128 (85.9%) |
| Doesn't blame anyone unless they are really sure about the problem | 28 (18.3%) | 22 (14.4%) | 103 (67.3%) | 9 (6%) | 10 (6.7%) | 130 (87.2%) |
| Sees if training to give staff more skills would help to fix the problem | 22 (14.4%) | 23 (15%) | 108 (70.6%) | 10 (6.7%) | 13 (8.7%) | 126 (84.6%) |
| Works with staff to plan how to fix the problem | 20 (13.1%) | 14 (9.2%) | 119 (77.8%) | 6 (4%) | 9 (6%) | 134 (89.9%) |

Table 6A Supervisors (a+b and a+b+c) “When counselling a troubled employee …”

| **Item** | **Baseline (N=68)** | | | **End-line (N=81)** | | |
| --- | --- | --- | --- | --- | --- | --- |
|  | **never/ rarely** | **some-times** | **usually/ always** | **never/ rarely** | **some-times** | **usually/ always** |
| I offer assistance when known problems or difficulties interfere with staff job performance | 2 (2.9%) | 4 (5.9%) | 62 (91.2%) | 0 (0%) | 1 (1.2%) | 80 (98.8%) |
| I listen, guide and encourage the employee to solve his/her own problems | 7 (10.3%) | 8 (11.8%) | 53 (77.9%) | 1 (1.2%) | 4 (4.9%) | 76 (93.8%) |
| I refer them to an appropriate support service if necessary | 4 (5.9%) | 5 (7.4%) | 59 (86.8%) | 1 (1.2%) | 3 (3.7%) | 77 (95.1%) |

**Table 6B Health Workers (a+b and a+b+c) “If I have problems, my supervisor…”**

| **Item** | **Baseline (N=153)** | | | **End-line (N=149)** | | |
| --- | --- | --- | --- | --- | --- | --- |
|  | **never/ rarely** | **some-times** | **usually/ always** | **never/ rarely** | **some-times** | **usually/ always** |
| Helps me if the problem is affecting how well I do my job | 17 (11.1%) | 22 (14.4%) | 114 (74.5%) | 9 (6%) | 12 (8.1%) | 128 (85.9%) |
| Listens to me and encourages me to solve my problem | 20 (13.1%) | 14 (9.2%) | 119 (77.8%) | 7 (4.7%) | 8 (5.4%) | 134 (89.9%) |
| Puts me in touch with a service that can help me, if this is what I need | 34 (22.2%) | 19 (12.4%) | 100 (65.4%) | 7 (4.7%) | 16 (10.7%) | 126 (84.6%) |

Table 7 Supervisor (a+b and a+b+c) time management…

| **Item** | **Baseline (N=68)** | | | **End-line (N=81)** | | |
| --- | --- | --- | --- | --- | --- | --- |
|  | **never/ rarely** | **some-times** | **usually/ always** | **never/ rarely** | **some-times** | **usually/ always** |
| I plan my daily, weekly and monthly schedule to allow time for the most important tasks… | 5 (7.4%) | 10 (14.7%) | 53 (77.9%) | 3 (3.7%) | 4 (4.9%) | 74 (91.4%) |
| I delegate tasks wherever possible | 1 (1.5%) | 2 (2.9%) | 65 (95.6%) | 1 (1.2%) | 4 (4.9%) | 76 (93.8%) |
| I ask my manager for advice when I have too much work and not enough time to carry out the supervision | 3 (4.4%) | 5 (7.4%) | 60 (88.2%) | 4 (4.9%) | 2 (2.5%) | 75 (92.6%) |

**Table 8A Supervisors – summary of total scores by intervention group**

| **Total score** | **Stage of data collection** | **INTERVENTION GROUP** | | | | | | | | |
| --- | --- | --- | --- | --- | --- | --- | --- | --- | --- | --- |
|  |  | **Control** | | | **a+b** | | | **a+b+c** | | |
|  |  | **Mean** | **SD** | **Median** | **Mean** | **SD** | **Median** | **Mean** | **SD** | **Median** |
| In my interactions with staff | Baseline | 21.56 | 2.12 | 21 | 21.81 | 2.52 | 22 | 22.19 | 2.79 | 23 |
|  | End-line | 21.3 | 2.58 | 21 | 22.4 | 2.62 | 23 | 22.8 | 2.23 | 23 |
| To maintain high levels of performance | Baseline | 37.37 | 3.7 | 37 | 35.34 | 4.53 | 35 | 37.83 | 6.69 | 39 |
|  | End-line | 36.44 | 5.22 | 36 | 38.83 | 5.28 | 39 | 39.59 | 5.44 | 42 |
| When dealing with performance problems | Baseline | 17.26 | 2.18 | 17 | 16.31 | 2.56 | 16 | 17.22 | 3.4 | 18 |
|  | End-line | 16.59 | 2.87 | 16 | 18.3 | 1.83 | 19 | 17.9 | 2.52 | 19 |
| Counselling a troubled employee | Baseline | 13.11 | 1.72 | 13 | 12.91 | 1.86 | 13 | 12.89 | 2.35 | 13.5 |
|  | End-line | 12.07 | 2.62 | 12 | 13.9 | 1.24 | 14 | 14 | 1.48 | 15 |
| Time management | Baseline | 12.63 | 2.1 | 13 | 12.75 | 2.05 | 13 | 13.5 | 2.08 | 15 |
|  | End-line | 12.52 | 2.23 | 13 | 13.9 | 1.39 | 14 | 13.61 | 2.12 | 15 |

Table 8B Health Workers - Summary of total scores by intervention group

| **Total Score** | **Stage of data collection** | **Control** | | | **Intervention A+B** | | | **Intervention A+B+C** | | |
| --- | --- | --- | --- | --- | --- | --- | --- | --- | --- | --- |
|  |  | **Mean** | **SD** | **Median** | **Mean** | **SD** | **Median** | **Mean** | **SD** | **Median** |
| How my supervisor interacts with staff - | Baseline | 20.51 | 4.6 | 22 | 20.61 | 3.86 | 21 | 20.6 | 4.5 | 22 |
|  | End-line | 19.74 | 4.02 | 20 | 21.52 | 3.06 | 21 | 22.16 | 3.32 | 23 |
| How my supervisor maintains high levels of performance | Baseline | 34.98 | 8.79 | 37 | 34.81 | 8.13 | 36 | 35.38 | 8.22 | 37 |
|  | End-line | 34.29 | 7.22 | 34 | 37.93 | 6.85 | 39 | 38.72 | 5.94 | 39 |
| How my supervisor deals with performance problems - sum of items | Baseline | 15.93 | 4.58 | 17 | 15.98 | 3.72 | 17 | 15.62 | 3.84 | 16 |
|  | End-line | 15.61 | 3.25 | 16 | 17.22 | 2.87 | 18 | 17.51 | 3.15 | 19 |
| If I have problems, my supervisor... - | Baseline | 12.09 | 3.42 | 13 | 11.63 | 3.21 | 12 | 12.18 | 3.32 | 13 |
|  | End-line | 11.61 | 2.91 | 12 | 12.74 | 2.64 | 13 | 13.16 | 2.14 | 14 |

**Table 9A Comparison of total scores at baseline and end-line (Supervisors)**

| **Total Score** | **Intervention group** | **Difference in sample means (end-line - baseline)** | **Standard error of the difference in sample means** | **Difference in sample medians (end-line - baseline)** | **Mann-Whitney U test** |
| --- | --- | --- | --- | --- | --- |
| In my interactions with staff | Control | -0.26 | 0.64 | 0 | U=344, Z=-0.36, p-value =0.72 |
|  | a+b | 0.59 | 0.61 | 1 | U=524, Z=-1.33, p-value=0.184 |
|  | a+b+c | 0.61 | 0.58 | 0 | U=661, Z=-0.8, p-value=0.424 |
| To maintain high levels of performance | Control | -0.93 | 1.23 | -1 | U=331, Z=-0.58, p-value =0.56 |
|  | a+b | 3.48 | 1.16 | 4 | U=373, Z=-3.03, **p-value=0.002** |
|  | a+b+c | 1.75 | 1.40 | 3 | U=624, Z=-1.17, p-value=0.243 |
| When dealing with performance problems | Control | -0.67 | 0.69 | -1 | U=310, Z=-0.95, p-value =0.34 |
|  | a+b | 1.99 | 0.54 | 3 | U=347.5, Z=-3.38, **p-value=0.001** |
|  | a+b+c | 0.68 | 0.69 | 1 | U=669.5, Z=-0.72, p-value=0.473 |
| Counselling a troubled employee | Control | -1.04 | 0.60 | -1 | U=285, Z=-1.41, p-value =0.159 |
|  | a+b | 0.99 | 0.38 | 1 | U=433.5, Z=-2.43, **p-value=0.015** |
|  | a+b+c | 1.11 | 0.46 | 1.5 | U=511.5, Z=-2.42, **p-value=0.015** |
| Time management | Control | -0.11 | 0.59 | 0 | U=356.5, Z=-0.14, p-value =0.888 |
|  | a+b | 1.15 | 0.42 | 1 | U=425.5, Z=-2.52, **p-value=0.012** |
|  | a+b+c | 0.11 | 0.48 | 0 | U=721, Z=-0.19, p-value=0.85 |

**Table 9B Change from baseline to end-line in total scores (Health Workers)**

| **Total scores** | **Intervention group** | **Difference in sample means (end-line - baseline)** | **Standard error of the difference in sample means** | **Difference in sample medians (end-line - baseline)** | **Mann-Whitney U test** |
| --- | --- | --- | --- | --- | --- |
| How my supervisor interacts with staff | Control | -0.15 | 0.19 | -0.4 | U=687, Z=-1.237, p-value=0.216 |
|  | a+b | 0.18 | 0.12 | 0 | U=1877, Z=-1.219, p-value =0.223 |
|  | a+b+c | 0.31 | 0.12 | 0.2 | U=2963.5, Z=-2.126, **p-value=0.033** |
| How my supervisor maintains high levels of performance | Control | -0.08 | 0.2 | -0.33 | U=731.5, Z=-0.811, p-value=0.418 |
|  | a+b | 0.34 | 0.15 | 0.33 | U=1663, Z=-2.206, **p-value =0.027** |
|  | a+b+c | 0.37 | 0.12 | 0.22 | U=2809, Z=-2.586, **p-value=0.01** |
| How my supervisor deals with performance problems | Control | -0.08 | 0.22 | -0.25 | U=685, Z=-1.262, p-value=0.207 |
|  | a+b | 0.3 | 0.15 | 0.25 | U=1757.5, Z=-1.788, p-value =0.074 |
|  | a+b+c | 0.48 | 0.13 | 0.75 | U=2373, Z=-3.976, **p-value=<0.001** |
| If I have problems, my supervisor... | Control | -0.16 | 0.23 | -0.33 | U=670, Z=-1.409, p-value=0.159 |
|  | a+b | 0.37 | 0.17 | 0.33 | U=1703, Z=-2.06, **p-value =0.039** |
|  | a+b+c | 0.33 | 0.14 | 0.34 | U=3180.5, Z=-1.474, p-value=0.14 |
