# Supplementary material for: The impact of a human resource management intervention on the capacity of supervisors to support and supervise their staff at health facility level
Source: Hum Resour Health. 2017 Aug 30;15:57. doi: 10.1186/s12960-017-0225-0 (PMC5577784; doi:10.1186/s12960-017-0225-0)
Supplement: Supplementary file 2 — Reliability statistics (Cronbach's alpha) for the adapted Supervisor Competency Self-Assessment Inventory (CSAI) in the health worker survey. (DOCX 13 bytes) [file 12960_2017_225_MOESM2_ESM.docx]

Additional file 2: Reliability statistics (Cronbach's alpha) for the adapted Supervisor Competency Self-Assessment Inventory (CSAI) in the health worker survey

| Supervisory activity | Number of items | Baseline | Endline |
| --- | --- | --- | --- |
| Interactions with staff | 5 | 0.869 | 0.896 |
| Maintaining high levels of performance | 9 | 0.915 | 0.921 |
| Dealing with performance problems | 4 | 0.83 | 0.881 |
| Counselling a troubled employee | 3 | 0.873 | 0.864 |
