# Supplementary material for: The impact of a human resource management intervention on the capacity of supervisors to support and supervise their staff at health facility level
Source: Hum Resour Health. 2017 Aug 30;15:57. doi: 10.1186/s12960-017-0225-0 (PMC5577784; doi:10.1186/s12960-017-0225-0)
Supplement: Supplementary file 3 — Reliability statistics (Cronbach's alpha) for the adapted Supervisor Competency Self-Assessment Inventory (CSAI) in the supervisor survey. (DOCX 13 bytes) [file 12960_2017_225_MOESM3_ESM.docx]

**Additional file 3: Reliability statistics (Cronbach's alpha) for the adapted Supervisor Competency Self-Assessment Inventory (CSAI) in the supervisor survey**

| **Supervisory activity** | **Number of items** | **Baseline** | **Endline** |
| --- | --- | --- | --- |
| Interactions with staff | 5 | 0.705 | 0.776 |
| Maintaining high levels of performance | 9 | 0.798 | 0.871 |
| Dealing with performance problems | 4 | 0.78 | 0.749 |
| Counselling a troubled employee | 3 | 0.621 | 0.778 |
| Time management | 3 | 0.723 | 0.656 |
